# Sprachverstehen im modulierten Störgeräusch bei bimodal versorgten CI-Tragenden

**DOI:** 10.1007/s00106-023-01323-9

**Published:** 2023-07-03

**Authors:** Tobias Weißgerber, Timo Stöver, Uwe Baumann

**Affiliations:** 1 Schwerpunkt Audiologische Akustik, Klinik für HNO-Heilkunde, Universitätsklinikum Frankfurt, Goethe-Universität Frankfurt, Theodor-Stern-Kai 7, 60590 Frankfurt am Main, Deutschland; 2 Klinik für HNO-Heilkunde, Universitätsklinikum Frankfurt, Goethe-Universität Frankfurt, Frankfurt am Main, Deutschland

**Keywords:** Sprachaudiometrie, Schwerhörigkeit, Hörimplantate, Hörstörungen, Hörhilfen, Speech audiometry, Hearing loss, Auditory implants, Hearing disorders, Hearing aids

## Abstract

**Hintergrund:**

Obwohl bei einer Versorgung mit Cochleaimplantaten (CI) ein gutes Sprachverstehen in Ruhe erreichbar ist, ist das Sprachverstehen im Störgeräusch gegenüber Normalhörenden (NH) stark beeinträchtigt. Bei einer bimodalen CI-Versorgung mit Hörgerät (HG) im Gegenohr beeinflusst das akustische Restgehör das Sprachverstehen im Störgeräusch.

**Fragestellung:**

Ziel der Arbeit war es, das Sprachverstehen im Störgeräusch bei bimodaler CI-Nutzung zu untersuchen und mit gleichaltrigen HG-Tragenden und Menschen ohne subjektive Hörminderung sowie einer jungen NH-Gruppe zu vergleichen.

**Material und Methoden:**

Es nahmen 19 bimodale CI-Tragende, 39 HG-Tragende und 40 subjektive Normalhörende der Altersklasse 60–90 Jahre und 14 junge NH teil. Die Sprachverständlichkeitsschwelle (SVS) im Störgeräusch wurde mit dem Oldenburger Satztest adaptiv für die 2 räumlichen Testkonditionen S0N0 (Sprache und Störgeräusch von vorne) und „multisource-noise field“ (MSNF, Sprache von vorne, 4 räumlich verteilte Störgeräuschquellen) jeweils im zeitlich kontinuierlichen Oldenburger Rauschen (Olnoise) und im zeitlich modulierten Fastl-Noise (amplitudenmoduliertes, sprachsimulierendes, fluktuierendes Störgeräusch nach Fastl) bestimmt.

**Ergebnisse:**

Mit zunehmender Hörminderung wurde die mediane SVS in allen Bedingungen signifikant schlechter. In der Testbedingung S0N0 war die SVS der CI-Gruppe im Olnoise um 5,6 dB und im Fastl-Noise um 22,5 dB schlechter als die der jungen NH-Gruppe (mittleres Alter: 26,4 Jahre) im MSNF betrugen die Unterschiede 6,6 dB (Olnoise) bzw. 17,3 dB (Fastl-Noise). In der jungen NH-Gruppe verbesserte sich die mediane SVS in der Bedingung S0N0 durch Lückenhören um 11 dB, in der älteren NH-Gruppe um nur noch 3,1 dB. In der HG-Gruppe und der bimodal versorgten CI-Gruppe gab es kein Lückenhören, und die SVS war im Fastl-Noise schlechter als im Olnoise.

**Schlussfolgerung:**

Bei fortschreitender Hörminderung wird das Sprachverstehen im modulierten Störgeräusch sogar stärker beeinträchtigt als im kontinuierlichen Störgeräusch.

## Abstract (en)

**Background:**

Although good speech perception in quiet is achievable with cochlear implants (CIs), speech perception in noise is severely impaired compared to normal hearing (NH). In the case of a bimodal CI fitting with a hearing aid (HA) in the opposite ear, the amount of residual acoustic hearing influences speech perception in noise.

**Objective:**

The aim of this work was to investigate speech perception in noise in a group of bimodal CI users and compare the results to age-matched HA users and people without subjective hearing loss, as well as with a young NH group.

**Materials and methods:**

Study participants comprised 19 bimodal CI users, 39 HA users, and 40 subjectively NH subjects in the age group 60–90 years and 14 young NH subjects. Speech reception thresholds (SRTs) in noise were adaptively measured using the Oldenburg Sentence Test for the two spatial test conditions S0N0 (speech and noise from the front) and multisource-noise field (MSNF; speech from the front, four spatially distributed noise sources) in continuous noise of the Oldenburg Sentence Test (Ol-noise) and amplitude-modulated Fastl noise (Fastl-noise).

**Results:**

With increasing hearing loss, the median SRT worsened significantly in all conditions. In test condition S0N0, the SRT of the CI group was 5.6 dB worse in Ol-noise than in the young NH group (mean age 26.4 years) and 22.5 dB worse in Fastl-noise; in MSNF, the differences were 6.6 dB (Ol-noise) and 17.3 dB (Fastl-noise), respectively. In the young NH group, median SRT in condition S0N0 improved by 11 dB due to gap listening; in the older NH group, SRTs improved by only 3.1 dB. In the HA and bimodal CI groups there was no gap listening effect and SRTs in Fastl-noise were even worse than in Ol-noise.

**Conclusion:**

With increasing hearing loss, speech perception in modulated noise is even more impaired than in continuous noise.

## Sprachverstehen bei Schwerhörigkeit

Bei Menschen mit ausgeprägten Innenohrschwerhörigkeiten, die mit konventionellen Hörgeräten nicht ausreichend versorgt werden können, ist die Versorgung mit einem Cochleaimplantat (CI) die Therapie der Wahl. Da häufig beide Ohren von Hörstörungen betroffen sind, wird i. d. R. im Gegenohr bei noch ausreichendem Versorgungserfolg ein Hörgerät (HG) genutzt (bimodale Versorgung) oder ansonsten bei Vorliegen der Indikationsvoraussetzungen (nach aktueller Leitlinie zur CI-Versorgung [1] bei einem Sprachverstehen von ≤ 60 %, gemessen mit dem Freiburger Einsilbertest [11] im freien Schallfeld bei 65 dB SPL), ebenfalls eine CI-Versorgung vorgenommen (bilaterale Versorgung). Unter ruhigen Hörbedingungen wird bei den meisten CI-Tragenden ein gutes Sprachverstehen erreicht. Allerdings kommt es in alltäglichen Hörumgebungen häufig zu einer Überlagerung des Sprachsignals durch Störgeräusche. Es ist bekannt, dass in solchen Situationen das Sprachverstehen von CI-Nutzern gegenüber Normalhörenden deutlich beeinträchtigt ist [18, 19]. Das gesunde auditorische System ist in der Lage, in Störgeräuschsituationen durch die Auswertung der Signalunterschiede beider Ohren die Informationen zu filtern und gewünschte Signale in der Wahrnehmung hervorzuheben (Cocktailparty-Effekt, [5]). Hierbei wird u. a. die räumliche Trennung der Störgeräuschquellen ausgenutzt. Außerdem weisen alltägliche Hintergrundgeräusche oftmals kurze zeitliche Pausen oder Lücken auf, die zu diesem Zeitpunkt den Signal-Rausch-Abstand verbessern und das Verstehen von Sprache bei Normalhörenden unterstützen („Lückenhören“, „glimpsing“, [6]). Rader et al. konnten bei bilateral CI-Tragenden und bimodal CI-Versorgten mit elektrisch-akustischer Stimulation (EAS) im Gegensatz zu Normalhörenden die Fähigkeit des Lückenhörens nicht nachweisen [19]. In der genannten Studie waren die Normalhörenden im Mittel jünger als die CI-Gruppen. Die Ergebnisse von Füllgrabe [10] zeigen jedoch, dass die zeitliche Verarbeitung mit zunehmendem Alter abnimmt, selbst wenn keine periphere Hörminderung vorliegt. Die Empfindlichkeit für zeitliche Feinstrukturen nahm sowohl bei monauralen als auch bei binauralen Hörversuchen mit zunehmendem Alter ab, und dies bereits ab der frühen Lebensmitte. Das Ziel der vorliegenden Arbeit war es, das Sprachverstehen im Störgeräusch in verschiedenen Hörumgebungen bei CI-Tragenden mit bimodaler Versorgung zu untersuchen und mit Gruppen von HG-Tragenden und subjektiv Normalhörenden der gleichen Altersklasse zu vergleichen. Insbesondere wurde der Einfluss des kontralateralen Ohrs mit HG auf die räumliche Entmaskierung („spatial release from masking“, SRM) und auf das Lückenhören untersucht.

## Material und Methoden

### Probanden

**Figure 1:**
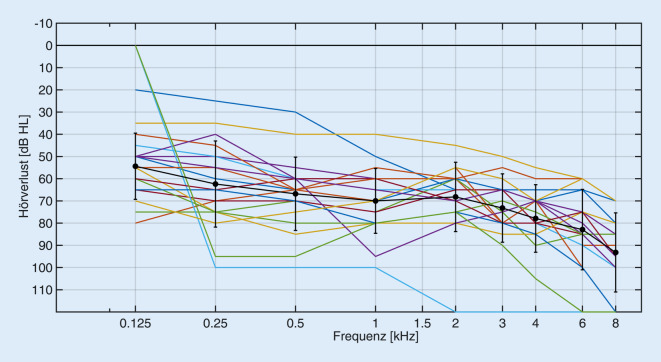

**Figure 2:**
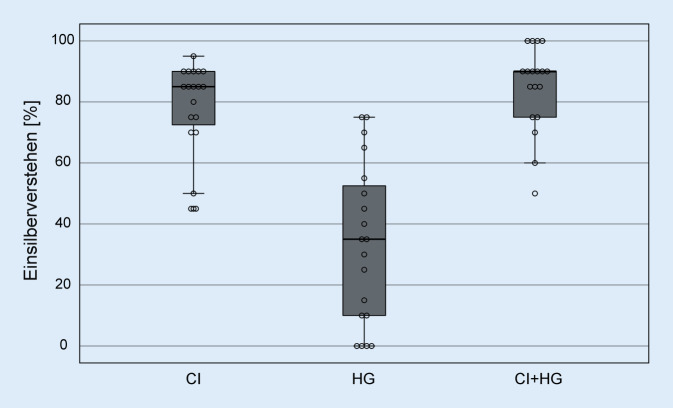

Das Probandenkollektiv der vorliegenden Studie umfasste insgesamt 19 Probanden (14 m., 5 w.) mit einem Alter von mindestens 60 Jahren. Deutliche Anzeichen für eine Demenz wurden mit einem Screening-Test (mindestens 9 Punkte im DemTect-Test, einem Testverfahren auf Demenzerkrankungen, [14]) ausgeschlossen. Die Probanden waren im Alter zwischen 61,2 und 84,0 Jahren (Durchschnittsalter ± Standardabweichung: 70,7 ± 6,2 Jahre). Jeder der Probanden war deutscher Muttersprachler. Alle Teilnehmenden waren einseitig mit einem CI versorgt (Fa. Cochlear, Macquarie, Australien) und nutzen im Gegenohr ein Hörgerät. Alle Implantate waren vom Typ CI24RE(CA) oder CI422, die genutzten Sprachprozessoren waren entweder CP810 oder CP910. Bei allen Tests wurde die Standardmikrofondirektionalität (Subniere) und die beim Teilnehmenden im Alltagsprogramm genutzte Dynamikverarbeitung, d. h. ggf. Autosensitivity Control™ (ASC)/Adaptive Dynamic Range Optimization (ADRO), genutzt, die zusätzliche Störgeräuschunterdrückung (SNR-NR) im Prozessor CP910 war immer deaktiviert. Die einzelnen Tonaudiogramme und das über alle Teilnehmenden gemittelte Tonaudiogramm des kontralateralen Ohrs sind in Abb. 1 dargestellt. Der mittlere Hörverlust (gemittelt über die Frequenzen 500, 1000, 2000 und 4000 Hz) betrug 70,3 ± 14,2 dB HL. Boxplots des Sprachverstehens in Ruhe (Freiburger Einsilbertest [11] bei 65 dB SPL im freien Schallfeld) sind seitengetrennt und beidohrig getestet in Abb. 2 dargestellt. Bei der Messung des Ohrs mit CI wurde das Gegenohr mit einem Ohrenstöpsel und zusätzlich mit einem ohrumschließenden Kapselgehörschutz geblockt. Das mittlere Einsilberverstehen auf dem mit CI versorgten Ohr betrug 77,9 ± 15,6 %, mit dem HG-Ohr 33,4 ± 26,6 % und beidohrig 84,7 ± 14,0 %.

Als Vergleichsgruppe diente eine Gruppe von 39 HG-Tragenden (3 mit HG rechts, 3 mit HG links, 33 mit HG beidseits; Durchschnittsalter: 76,0 ± 4,7 Jahre, weitere demografische Daten in [15]), eine Gruppe von 40 Menschen ohne subjektiven Hörverlust (Durchschnittsalter: 69,3 ± 7,1 Jahre) sowie eine junge Kontrollgruppe Hörgesunder (*n* = 14, Durchschnittsalter: 26,4 ± 5,4 Jahre). Die Ergebnisse zum Sprachverstehen im Störgeräusch der Gruppe ohne subjektiven Hörverlust und junger Hörgesunder sind der Publikation von Weißgerber et al. [25] entnommen.

Die Studie wurde von der Ethikkommission des Fachbereichs Medizin der Johann Wolfgang Goethe-Universität in Frankfurt am Main unter der Geschäftsnummer 164/13 genehmigt.

### Messung des Sprachverstehens im Störgeräusch

Die Messungen erfolgten in einem reflexionsarmen Raum mit den Dimensionen 4,1 m x 2,6 m x 2,1 m (Länge × Breite × Höhe). Das Wiedergabesystem bestand aus 128 unabhängigen Lautsprecherkanälen, die in Rechteckanordnung raumumschließend in der Horizontalebene auf einer Höhe von 1,20 m angeordnet waren. Durch das Wiedergabeverfahren der Wellenfeldsynthese (WFS) [2] ist es mit diesem System möglich, virtuelle Schallquellen in fast jeder beliebigen Position innerhalb oder auch außerhalb des Raums zu schaffen. Weitere detaillierte Informationen zum Wiedergabesystem sind von Weißgerber [24] beschrieben.

Mit dem Oldenburger Satztest (OlSa, [21–23]) wurde die Sprachverständlichkeitsschwelle (SVS) für 50 % Sprachverstehen im Störgeräusch ermittelt. Der Störgeräuschpegel war konstant bei einem Schalldruckpegel von 65 dB, der Sprachpegel wurde nach dem Verfahren von Brand und Kollmeier [3] adaptiv entsprechend der Anzahl an korrekt erkannten Wörtern angepasst.

Der Test erfolgte im geschlossenen Antwortmodus, d. h. der Proband befand sich allein im Prüfraum und musste auf einem Touchscreen nach Hören des Satzes die Elemente des Satzes durch Berühren der entsprechenden Wortfelder auf dem Touchscreen auswählen, die er verstanden hatte. Vor Beginn der Studientests wurde mit jedem Teilnehmenden ein Trainingsdurchlauf in Ruhe bei festem Sprachpegel von 65 dB SPL und eine adaptive Testliste (beides jeweils 30er-Testlisten) im Störgeräusch durchgeführt. Im Anschluss daran erfolgten 4 Durchgänge des OlSa mit 20er-Testlisten in zufälliger Reihenfolge. Es wurden 2 verschiedene räumliche Konfigurationen von Sprach- und Störschall mit 2 verschiedenen Störgeräuscharten untersucht.

Bei den beiden Störgeräuschen handelte es sich zum einen um das zeitlich kontinuierliche Oldenburger Rauschen (Olnoise), dessen Langzeitspektrum mit dem des OlSa-Wortmaterials übereinstimmt [21]. Zum anderen wurde das amplitudenmodulierte, sprachsimulierende, fluktuierende Störgeräusch nach Fastl (Fastl-Noise) [8] verwendet. Die spektrale Verteilung der Amplitudenmodulation erreicht ein Maximum bei einer Modulationsfrequenz von 4 Hz, was in etwa der mittleren Silbengeschwindigkeit westlicher Sprache entspricht [9].

Die beiden räumlichen Testkonfigurationen waren S0N0 und das „multisource-noise field“ (MSNF) nach Rader et al. [19]. Bei S0N0 wurden Sprachsignal (S) und Störgeräusch (N) aus gleicher Richtung von 0° frontal mit einem Abstand von 1,75 m zum Probanden dargeboten. Hierzu wurden 4 benachbarte Lautsprecher des Wiedergabesystems genutzt, um einen ausreichenden Schalldruckpegel an der Position des Probanden zu erhalten [26].

In der Testkondition MSNF wurde das Sprachsignal ebenfalls aus den Frontlautsprechern in 0°-Position mit einem Abstand von 1,75 m zum Probanden erzeugt. Durch WFS wurde ein diffuses Störschallfeld mit 4 virtuellen Störgeräuschquellen an den Positionen ±28,6° und ±151,4° mit einem Abstand von 1,25 m zur Mitte des Kopfs des Probanden erstellt [26]. Die 4 virtuellen Störgeräuschquellen gaben das Störgeräusch zeitlich dekorreliert wieder. Die Lautsprecherkonfiguration MSNF wurde ausgewählt, um alltägliche Gesprächssituationen in lautem Umfeld, wie beispielsweise Gespräche in einem Restaurant, zu simulieren.

### Statistik

Die erhobenen Daten und Messwerte wurden mit dem Statistikprogramm SPSS 27 (Fa. IBM, Armonk, NY, USA) verarbeitet und ausgewertet. Die Zielgrößen der verschiedenen Tests wurden mit dem Shapiro-Wilk-Test auf Normalverteilung überprüft. Da keine der Zielgrößen eine Normalverteilung aufwies, erfolgte die weitere Auswertung ausschließlich mit nichtparametrischen Verfahren. Zur Überprüfung der Ergebnisunterschiede auf Signifikanzen wurde bei 2 unverbundenen Stichproben der Mann-Whitney-U-Test (Prüfgröße: *Z*_*u*_) und bei mehr als 2 unverbundenen Stichproben der Kruskal-Wallis-Test (Prüfgröße: *H*) angewendet. Handelte es sich um 2 verbundene Stichproben, wurde der Wilcoxon-Test (Prüfgröße: *Z*_*w*_) angewendet. Bei mehrfachen Paarvergleichen wurde das Signifikanzniveau korrigiert (Verfahren nach Benjamini-Hochberg).

## Ergebnisse

**Figure 3:**
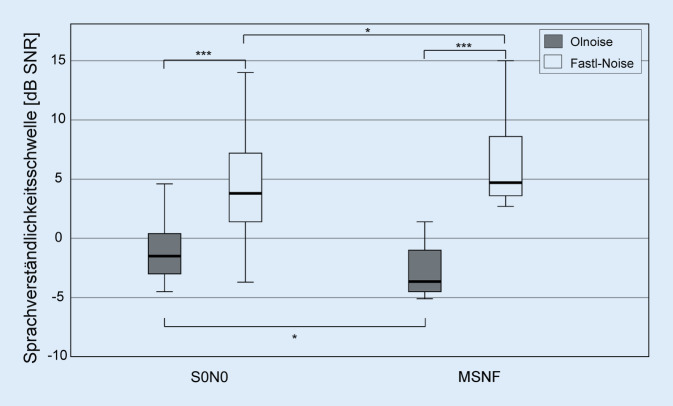

In Abb. 3 sind die Ergebnisse der bimodalen CI-Gruppe für die beiden Testbedingungen S0N0 und MSNF jeweils für die Störgeräusche Olnoise und Fastl-Noise dargestellt. Unter der Testbedingung S0N0 betrug die mediane SVS in der Störgeräuschkondition Olnoise −1,5 dB SNR (Signal-to-Noise-Ratio), im Fastl-Noise wurde eine SVS von 4,5 dB SNR erreicht. Die Ergebnisse im Fastl-Rauschen waren signifikant um 6 dB schlechter als im Olnoise (*Z*_*w*_ = −3,724; *p* < 0,001). In der Testbedingung MSNF wurde in der Störschallbedingung Olnoise eine mediane SVS von −3,5 dB SNR, in der Störschallbedingung Fastl-Noise eine SVS von 4,8 dB SNR erreicht. Die Ergebnisse im Fastl-Rauschen waren auch hier signifikant schlechter (8,3 dB, *Z*_*w*_ = −3,823; *p* < 0,001).

Für das Olnoise wurde ein signifikanter Einfluss der räumlichen Präsentationsart (SRM) von 2 dB festgestellt (*Z*_*w*_ = −2,593; *p* = 0,013), die Verschlechterung der SVS im Fastl-Rauschen im MSNF in Höhe von 0,3 dB war ebenfalls statistisch signifikant (*Z*_*w*_ = −2,496; *p* = 0,013).

**Figure 4:**
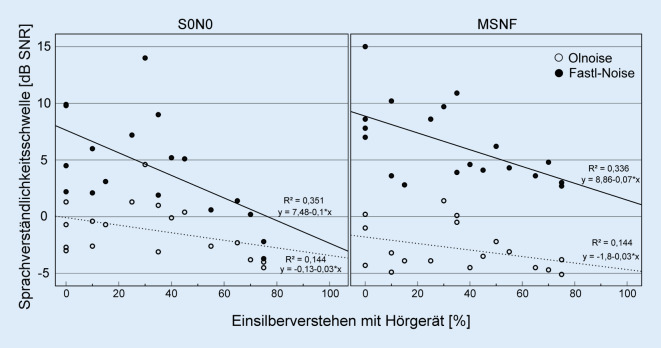

Unter der Testbedingung S0N0 zeigte sich eine signifikante Korrelation des mittleren Hörverlusts der hörgeräteversorgten Seite mit der SVS (Olnoise: *ρ* = 0,49; *p* = 0,037; Fastl-Noise: *ρ* = 0,40; *p* = 0,039). Für die SVS im Fastl-Rauschen zeigte sich eine signifikante negative Korrelation mit dem Einsilberverstehen mit Hörgerät (Streu-Punkt-Diagramme in Abb. 4, S0N0: *ρ* = 0,61; *p* = 0,006; MSNF: *ρ* = 0,57; *p* = 0,012). Zwischen SVS und Einsilberverstehen mit CI konnte keine Korrelation festgestellt werden.

**Figure 5:**
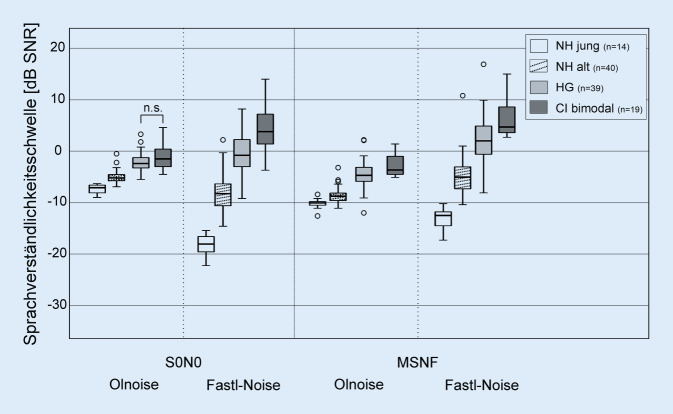

Ein Vergleich der Ergebnisse der bimodalen CI-Gruppe mit gleichaltrigen Gruppen mit und ohne Hörgerät sowie mit einer jungen Normalhörenden Gruppe ist in Abb. 5 dargestellt. Für alle Testbedingungen zeigte sich ein signifikanter Einfluss der Probandengruppe (S0N0 Olnoise: *H* = 72,911; S0N0 Fastl-Noise: *H* = 81,744; MSNF Olnoise: *H* = 74,054; MSNF Fastl-Noise: *H* = 80,475; alle *df* = 3, alle *p* < 0,001). Im Vergleich der Gruppen hatten solche mit stärker ausgeprägter Hörminderung im Median schlechtere SVS als Gruppen mit geringerer oder keiner Hörminderung (d. h. NH jung → NH alt → HG-Nutzer → Bimodal CI). Lediglich in der Kondition S0N0 konnte im Olnoise kein Unterschied in der SVS zwischen der HG- und der CI-Gruppe festgestellt werden.

Unter der Testbedingung S0N0 war die SVS der CI-Gruppe im Olnoise um 5,6 dB und im Fastl-Noise um 22,5 dB schlechter als die der jungen NH-Gruppe, im MSNF betrugen die Unterschiede 6,6 dB (Olnoise) bzw. 17,3 dB (Fastl-Noise). Im Vergleich zur Gruppe der HG-Tragenden war die SVS im Olnoise nur um 0,9 dB (S0N0) bzw. 1,2 dB (MSNF) schlechter, während im Fastl-Noise die Differenz im Mittel 5,3 dB (S0N0) bzw. 2,8 dB (MSNF) betrug.

In der jungen NH-Gruppe verbesserte sich die SVS in der Bedingung S0N0 durch Lückenhören im Mittel um 11 dB (*Z*_*w*_ = −3,297; *p* < 0,001), in der älteren NH-Gruppe um nur noch 3,1 dB (*Z*_*w*_ = −5,216; *p* < 0,001). In der Gruppe mit HG-Versorgung führte das Fastl-Rauschen im Vergleich zum Olnoise wie in der bimodalen CI-Gruppe zu einer Verschlechterung der SVS (1,6 dB; *Z*_*w*_ = −2,916; *p* = 0,004).

In der jungen NH-Gruppe verbesserte sich die SVS in der Bedingung MSNF durch Lückenhören im Mittel um 2,4 dB (*Z*_*w*_ = −3,297; *p* < 0,001). In der älteren NH-Gruppe und in der Gruppe mit HG-Versorgung führte das Fastl-Rauschen im Vergleich zum Olnoise wie in der bimodalen CI-Gruppe zu einer Verschlechterung der SVS (NH alt: 3,7 dB; *Z*_*w*_ = −5,276; *p* < 0,001; HG: 6,7 dB; *Z* = −5,443; *p* < 0,001).

Alle Probandengruppen zeigten im Olnoise ein etwa vergleichbares SRM (Differenz der medianen SVS von S0N0 und MSNF) zwischen 2 und 3,6 dB.

## Diskussion

Es wurden die Sprachverständlichkeitsschwellen im Störgeräusch von 3 im Mittel gleichaltrigen (älter als 60 Jahre) Probandengruppen (NH, HG-Tragende, bimodale CI-Tragende) und von einer jungen NH-Gruppe für die beiden räumlichen Testkonditionen S0N0 und MSNF im kontinuierlichen Olnoise und im zeitlich modulierten Fastl-Noise verglichen. Mit zunehmendem Hörverlust verschlechterte sich die SVS in allen Testbedingungen. Während sich im kontinuierlichen Olnoise die SVS der HG- und bimodalen CI-Gruppe nur geringfügig unterscheiden, ist die SVS der bimodalen CI-Gruppe im modulierten Fastl-Noise deutlich schlechter als in allen anderen Gruppen.

### Einfluss des Störgeräuschs auf das Sprachverstehen

Der Einfluss des Störgeräuschs auf das Sprachverstehen, also der Effekt des Lückenhörens, wurde in der jungen NH-Gruppe in beiden räumlichen Störgeräuschkonditionen S0N0 und MSNF nachgewiesen und war hier in derselben Größenordnung (S0N0: 11 dB, MSNF: 2,4 dB) wie von Rader et al. [19] beschrieben. In der älteren NH-Gruppe (mit altersbedingtem Hörverlust) war das Lückenhören in S0N0 mit nur noch 3,1 dB bereits um 7,9 dB schlechter als in der jungen NH-Gruppe. In der HG-Gruppe und der bimodal versorgten CI-Gruppe gab es keinen Effekt des Lückenhörens. Im Gegenteil war die SVS im modulierten Störgeräusch sogar schlechter als im kontinuierlichen Olnoise. Diese Ergebnisse stimmen mit vorherigen Studien unter Einsatz gleicher Störgeräusche mit bimodaler EAS bzw. bilateralem CI [19] oder bilateralem CI [26] überein. Auch Hey et al. [12] berichten über Lückenhören bei NH in einem modulierten ICRA7-Rauschen (6 überlagerte Sprecher, d. h. weniger moduliert als das Fastl-Noise), während bei CI-Tragenden im Alter zwischen 43–80 Jahren die SVS sich im Mittel um 4 dB verschlechterte. In einer weiteren Arbeit von Hey et al. [13] wird ebenfalls über starke Defizite im Lückenhören bei CI-Tragenden im Vergleich zu NH (mehr als 20 dB schlechtere SVS) berichtet. Zirn et al. [27] haben in einer Gruppe von CI-Tragenden (unilateral, bimodal und bilateral gemischt) mit einem Alter unter 65 Jahren bei S0N0 im Fastl-Noise ebenfalls eine Verschlechterung der SVS festgestellt. Lediglich in der Arbeit von Weißgerber et al. [26] wurde in der bimodalen CI-Gruppe (Altersdurchschnitt: 49,6 ± 19,1 Jahre) ein geringer Effekt des Lückenhörens festgestellt. In der vorliegenden Arbeit konnte dieser Effekt zwar nicht bestätigt werden, es zeigte sich in der bimodalen CI-Gruppe jedoch eine negative Korrelation des Sprachverstehens der HG-versorgten Seite mit der SVS im Fastl-Noise, welche unter der Olnoise-Bedingung nicht vorhanden war. Mit zunehmender kontralateraler akustischer Hörfähigkeit erhöht sich also bei bimodaler CI-Versorgung die Möglichkeit des Lückenhörens.

Neben dem eingeschränkten Dynamikbereich in der CI- und HG-Gruppe und der relativ geringen Frequenzauflösung in CI-Systemen gibt es auch bereits bei zunehmendem Alter und nur leichtgradigen Hörminderungen Defizite bei der Frequenzselektion, die zu einer schlechteren Trennung von Sprache und Geräuschen führen und es schwieriger oder unmöglich machen, zeitliche Lücken zu erkennen [16]. Duquesnoy beschreibt, dass ältere Personen im Alter von 75–88 Jahren mit Presbyakusis weniger in der Lage sind, die zeitlichen Lücken in fluktuierendem Lärm zu nutzen als normalhörende Personen [7]. Peters et al. zeigten, dass sowohl das Alter als auch die Hörminderung einen erheblichen Einfluss auf das Sprachverstehen haben, wobei die Auswirkungen bei moduliertem Störgeräusch am größten sind [17]. Bei jungen normalhörenden Erwachsenen betrug die Verbesserung der SVS durch das Lückenhören 4–7 dB, während bei älteren Personen mit Hörverlust nur eine Verbesserung um 1,5 dB festgestellt wurde. Die Ergebnisse von van Summers und Molis [20] deuten darauf hin, dass die Hörbarkeit von Signalen nicht der Hauptfaktor ist, der das Lückenhören bei einer leichten bis mittelschweren Hörminderung einschränkt. Vielmehr ist die verringerte Fähigkeit des Ausnutzens von zeitlichen Fluktuationen im Maskierer bei der Mehrheit der Studienteilnehmer selbst bei einer Erhöhung des Präsentationspegels um bis zu 30 dB weiterhin vorhanden. Eher könnten Einschränkungen bei der Verarbeitung von überschwelliger Sprache für das verminderte Lückenhören verantwortlich sein, z. B. eine mögliche Verschlechterung der zeitlichen Auflösung. Die Ergebnisse von Füllgrabe zeigen, dass die zeitliche Verarbeitung mit zunehmendem Alter abnimmt, auch wenn keine periphere Hörminderung vorliegt [10]. Die Empfindlichkeit für zeitliche Feinstruktur nahm sowohl bei monauralen als auch bei binauralen Hörversuchen mit zunehmendem Alter ab, und zwar bereits ab der frühen Lebensmitte. Daher ist bei Gruppenvergleichen zur Fähigkeit des Lückenhörens auch immer das Alter der verschiedenen Gruppen zu berücksichtigen. In der vorliegenden Studie war das Alter der 3 Vergleichsgruppen ohne und mit HG sowie mit bimodaler CI-Versorgung möglichst gut angeglichen, und es zeigten sich im Demenzscreening auch keine signifikanten Gruppenunterschiede.

### Einfluss der räumlichen Testbedingung

Die Rolle der binauralen Verarbeitung zeigt sich im Vergleich der Testbedingung S0N0 mit dem MSNF. Der Einfluss der räumlichen Testbedingung („spatial release from masking“, SRM) führt hier aufgrund des Kopfschatteneffekts und des binauralen Squelch-Effekts [4] zu verbesserten SRT im Vergleich zur S0N0-Bedingung. In der Studie von Duquesnoy [7] wurde festgestellt, dass das SRM bei einem Rauschsignal von der Seite und dem Sprachsignal von vorne gegenüber S0N0 bei jungen Normalhörenden 5–9 dB und bei älteren Personen mit altersbedingtem Hörverlust 3–4 dB beträgt.

In der vorliegenden Studie war das SRM im MSNF bei kontinuierlichem Olnoise in allen Probandengruppen mit 2–3,5 dB vergleichbar ausgeprägt. Die Ergebnisse zum SRM der bimodalen CI-Gruppe sind mit bisherigen Ergebnissen im gleichen Test-Setup mit bimodalen und bilateralen CI-Tragenden (etwa 2 dB SRM in einer Arbeit von Weißgerber et al. [26]) sowie mit bimodal EAS bzw. bimodal CI (etwa 3 dB SRM in einer Arbeit von Rader et al. [19]) vergleichbar.

In der MSNF-Bedingung mit Fastl-Noise ist der Effekt des SRM nicht einzeln zu beurteilen, da hier zusätzlich zur Änderung der räumlichen Konfiguration auch (monaural gesehen) die Modulationseigenschaften (4 unkorreliert überlagerte Quellen mit Fastl-Noise bei MSNF vs. eine Quelle Fastl-Noise bei S0N0) des Störgeräuschs verschieden sind. Obwohl die Störgeräusche räumlich vom Zielsprecher getrennt sind und zusätzlich noch zeitliche Lücken im Störgeräusch vorhanden sind, werden in allen 3 älteren Probandengruppen in diesen Testbedingungen im Vergleich zu allen anderen Testbedingungen die schlechtesten SVS erreicht, was sowohl auf einen kombinierten Effekt der verschlechterten binauralen als auch zeitlichen Verarbeitung zurückzuführen sein könnte.

## Fazit für die Praxis

- Um die Hörleistung von Menschen mit Hörminderung in alltäglichen Hörbedingungen besser beurteilen zu können, ist der Einsatz von zeitlich fluktuierenden Störgeräuschen in der Sprachaudiometrie zu empfehlen.
- Darüber hinaus sollte eine Testbedingung mit räumlich aufgetrennter Störgeräuschbedingung verwendet werden.
- Selbst bei nur leichtgradigem Hörverlust nimmt die Lückenhörfähigkeit mit zunehmendem Alter ab.
- Bei fortschreitender Hörminderung mit Hörgerät(HG)- oder Cochleaimplantat(CI)-Versorgung wird das Sprachverstehen im modulierten Störgeräusch sogar stärker beeinträchtigt als im kontinuierlichen Störgeräusch.
- Im kontinuierlichen Störgeräusch ist die Fähigkeit der räumlichen Entmaskierung („spatial release from masking“, SRM) in allen untersuchten Gruppen vergleichbar ausgeprägt vorhanden.
